# Editorial: Current Status and the Need for Acute and Chronic Modulation of Brain Circuits as Interventions in Neurological and Psychiatric Disorders

**DOI:** 10.3389/fnhum.2022.927382

**Published:** 2022-06-27

**Authors:** Zheng Z. Wei, Bin Qiu, Xiaopeng Song, Yuxuan Liu

**Affiliations:** ^1^Department of Neurology, Beijing Friendship Hospital Center for Neurological Disorders, Beijing, China; ^2^Department of Pharmacology, Yale School of Medicine, New Haven, CT, United States; ^3^McLean Imaging Center, McLean Hospital, Harvard Medical School, Belmont, MA, United States; ^4^Department of Pediatrics, Stanford University, Stanford, CA, United States

**Keywords:** brain protection, cerebrovascular disease, clinical neuroscience, neurocritical care, neuroimaging

In neurocritical care, severe neuroinflammatory conditions and systematic inflammatory storms can occur in central nervous system infection-induced encephalitis, meningitis, or pyogenic brain infections. Systematic inflammatory conditions are observed in severe infection-associated thrombosis including COVID-19 and immunotherapy against tumors. In addition, 20–30% of patients in the ICU develop neurological/psychiatric manifestations during their recovery. These pathological conditions can lead to an even larger number of long-term sleep disorder and cognitive deficit cases (Kamdar et al., 2013; Song et al., 2021).

Given the critical role of pathological changes of brain circuits in detrimental neurological/psychiatric disorders, various treatments have been tested. Their acute/chronic diseased/pathobiological conditions can be evidenced by recent progress. For instance, in long-term changes of depression, the relationship between depression severity and amygdala subregion volume deserves to be studied further (Tesen et al., 2022). Primary evidence was provided by the authors when studying first-episode and drug-naïve patients. The use of drugs that treat chronic insomnia might increase significant long-term mild cognitive impairment along with brain region changes. The authors and many other researchers support the fact that benzodiazepine receptor agonists (zolpidem, zaleplon, zopiclone) are effective (Guo et al., 2021) but more studies about long-term effects are underway. In neurocritical care patients, the authors test respiratory outcomes, including the incidence of pneumonia, mechanical ventilation, sputum viscosity, and their beneficial effects of the level of consciousness, motor, and verbal response improvement (Wang et al., 2022). Other researchers have investigated the specific scaling method for measuring the acute exercise-induced fatigue to help managing patients with chronic fatigue better, which may also provide clinical significance for the treatment in neurological or psychiatric diseases. In addition to neurological recovery, cognitive and mood disorders that develop at later stages are followed up in the study design.

Chronic fatigue syndrome involves experiences of persistent fatigue in patients, whose clinical presentation includes mild cognitive impairment (usually characterized as central fatigue) in addition to physical changes such as peripheral fatigue. The authors, including their collaborators from the China Institute of Sports Medicine, have investigated the specific scaling method for measuring acute exercise-induced fatigue (Lu et al., 2022). They may also provide clinical significance for treatment in chronic diseases of cancer, diabetes, obesity, and heart diseases. In our recent research protocols, long-distance runners are tested using their scale to evaluate chronic fatigue status. Additional benefits may include grading of exercise-related central/peripheral fatigue, retest consistency with cognitive impairment, and the self-evaluation of cognitive fatigue.

Neuromodulation facilitates the interactions of neuroinflammatory factors and microenvironment improvement and is therapeutically promising. One of the methods under neuromodulation of neuronal circuit activity targets a cellular mechanism representing the emerging field as a new potential therapeutic strategy (Figure 1). In addition, many brain stimulation methods can be combined with current electrophysiology or magnetoencephalography for spontaneous or evoked electromagnetic brain activity monitoring, neuroimaging like fMRI/MRI, or systems pharmacology analyses. Our recent research editions provide systematic reviews on the neuroprotection/repair mechanisms involved in neurocritical care (from clinically managing acute stroke, cerebral infection, cerebrovascular spasm, and neuroinflammation) based on neuroimaging analysis, as well as pharmacological approaches with neurological/psychiatric assessments in neuroprotection for patients affected by those disorders. We here emphasize that neuromodulation (such as photobiomodulation) is durable, which is tested in clinical investigations from our group and some others (Li et al., 2021). The stimulation can be performed at an early stage of brain involvement in chronically diseased state and recovery stages following acute diseases and delayed disorders.

**Figure 1:**
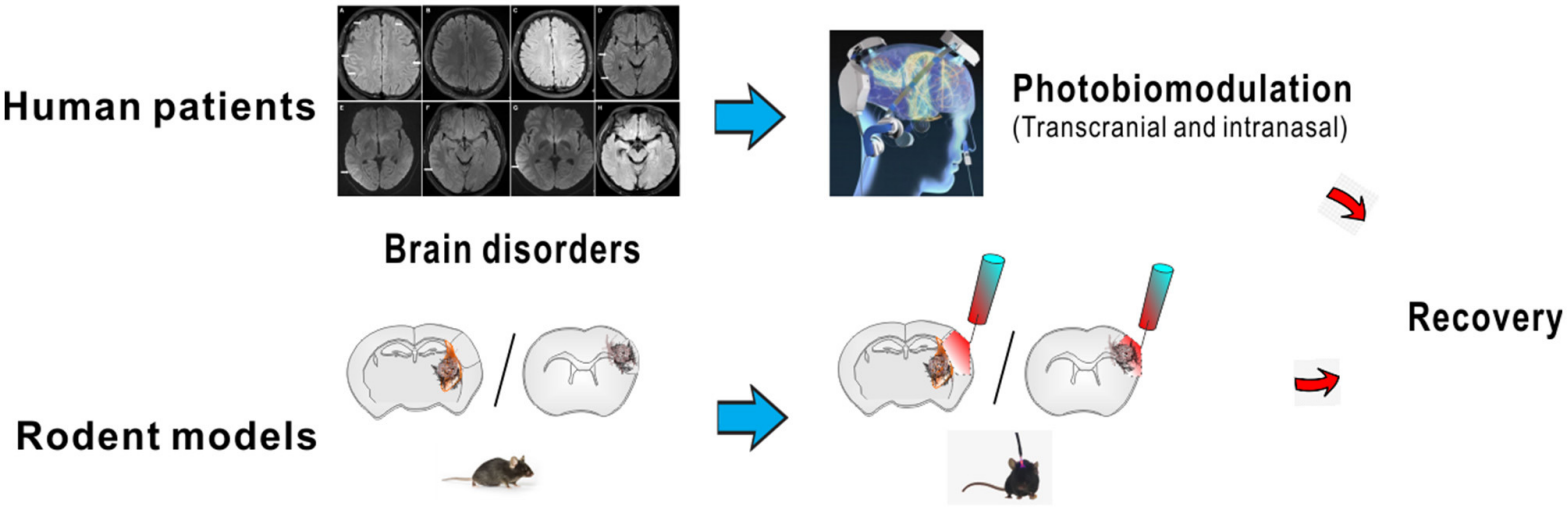
Photobiomodulation as potential treatment in brain disorders. The stimulation could be performed at an early stage of brain involvement such as for chronic brain disorders. It facilitated the recovery at later stages from acute brain diseases at delayed treatments. The graph was partially adopted from the collection (Shu et al., 2022). The brain imaging image was proposed.

## Author Contributions

All authors listed have made a substantial, direct, and intellectual contribution to the work and approved it for publication.

## Funding

All potential funding supports for this project were from Beijing Association for Science and Technology, and from the American Heart Association/the American Stroke Association.

## Conflict of Interest

The authors declare that the research was conducted in the absence of any commercial or financial relationships that could be construed as a potential conflict of interest.

## Publisher's Note

All claims expressed in this article are solely those of the authors and do not necessarily represent those of their affiliated organizations, or those of the publisher, the editors and the reviewers. Any product that may be evaluated in this article, or claim that may be made by its manufacturer, is not guaranteed or endorsed by the publisher.
